# Development and Validation of an Extractive Spectrophotometric Method for Miconazole Nitrate Assay in Pharmaceutical Formulations

**DOI:** 10.1155/2018/2191072

**Published:** 2018-04-17

**Authors:** Tadele Eticha, Getu Kahsay, Teklebrhan Hailu, Tesfamichael Gebretsadikan, Fitsum Asefa, Hailekiros Gebretsadik, Boovizhikannan Thangabalan

**Affiliations:** School of Pharmacy, College of Health Sciences, Mekelle University, Mekelle, Ethiopia

## Abstract

A simple extractive spectrophotometric technique has been developed and validated for the determination of miconazole nitrate in pure and pharmaceutical formulations. The method is based on the formation of a chloroform-soluble ion-pair complex between the drug and bromocresol green (BCG) dye in an acidic medium. The complex showed absorption maxima at 422 nm, and the system obeys Beer's law in the concentration range of 1–30 *µ*g/mL with molar absorptivity of 2.285 × 10^4^ L/mol/cm. The composition of the complex was studied by Job's method of continuous variation, and the results revealed that the mole ratio of drug : BCG is 1 : 1. Full factorial design was used to optimize the effect of variable factors, and the method was validated based on the ICH guidelines. The method was applied for the determination of miconazole nitrate in real samples.

## 1. Introduction

Miconazole nitrate, chemically 1-[(2RS)-2-[(2,4-dichlorobenzyl)oxy]-2-(2,4-dichlorophenyl)ethyl]-1H-imidazole nitrate, is an antifungal azole [1]. It is one of the most commonly used topical azoles and available over the counter. It is used for vulvovaginal candidiasis and dermatophytic infections, including tinea corporis, tinea pedis, and tinea cruris [2].

Several analytical methods have been reported for the determination of miconazole in biological samples, pure and pharmaceutical dosage forms using various chromatographic methods such as high-performance liquid chromatography (HPLC) [3–7], gas chromatography (GC) [6, 8, 9], high-performance thin-layer chromatography (HPTLC) [10], hyphenated technique (gas chromatography-mass spectroscopy, GC-MS) [11], and spectrophotometry [12–14]. The official pharmacopoeial methods such as the United States Pharmacopeia (USP) and British Pharmacopoeia (BP) have recommended HPLC for miconazole nitrate assay in pharmaceutical preparations [1, 15].

Extractive spectrophotometric techniques are popular for their sensitivity in the quantification of pharmaceutical compounds. Hence, considerable attention has been given to ion-pair extractive spectrophotometric methods for the assay of many drugs [16–18]. The objective of the present study was to develop a simple, less time-taking, and cheap extractive spectrophotometric method for routine analysis of miconazole nitrate in pharmaceutical preparations.

## 2. Experimental

### 2.1. Apparatus

All absorption spectra were made using a double beam UV-Vis spectrometer (PG Instruments, Lutterworth, England), which is equipped with 1 cm matched quartz cells, connected to a computer loaded with UVWin PC software, and used for all the absorbance measurements and data manipulation. A pH meter (Adwa Instruments, Romania) was employed to measure the pH of buffer solutions.

### 2.2. Reagents and Samples

All chemicals used were of analytical reagent grade. Chloroform (Loba Chemie, Mumbai, India) and ethanol 98% (Dallul Pharmaceutical PLC, Addis Ababa, Ethiopia) were employed, whereas distilled water was used throughout the work. Pharmaceutical grade of miconazole was obtained from Addis Pharmaceutical Factory (Adigrat, Ethiopia). Freshly prepared solutions were always employed. Commercial samples containing miconazole nitrate were purchased from the local pharmacy.

### 2.3. Preparation of Standard Solutions

A standard solution of a drug (200 *μ*g/mL) was prepared by dissolving miconazole nitrate in 98% ethanol. Bromocresol green (BCG) solution (200 *μ*g/mL) was prepared by dissolving BCG in 10 mL ethanol and diluted to 100 mL with distilled water, while a buffer solution was prepared from potassium hydrogen phthalate in water.

### 2.4. Procedure for Miconazole Nitrate Assay

Miconazole nitrate standard solution of 1 mL was pipetted into a 10 mL volumetric flask. Six millilitres of BCG (200 *μ*g/mL) and 2 mL buffer of pH 4 were added and diluted up to the mark with distilled water followed by mixing well. The solution was transferred to a separatory funnel, and it was shaken with 10 mL chloroform for 2 minutes and then allowed to stand for clear separation of the two phases. The chloroform layer was passed through anhydrous sodium sulphate; the chloroform extracts were collected and diluted to 10 mL with chloroform. The absorbance of the yellow-colored complexes was measured at 422 nm against a reagent blank.

### 2.5. Preparation of Pharmaceutical Samples

A cream dosage form, miconazole nitrate cream equivalent to 20 mg of the drug, was weighed and transferred into a 50 mL volumetric flask. A soft gelatin capsule, the miconazole nitrate vaginal soft gelatin capsule, was weighed, and the content was transferred into a flask. The weight of the contents was obtained by taking the difference in weights of the intact capsule and washed shell. The content equivalent to 20 mg of the drug was weighed and transferred into a 50 mL volumetric flask. The drug taken from both dosage forms was dissolved in ethanol with gentle heating and diluted to the mark. The solution was appropriately diluted and assayed by the proposed method. The validity of the method was confirmed by applying the standard addition technique.

### 2.6. Experimental Design

Optimization of the method was performed by experimental design and factorial analysis of variance using SPSS statistical package version 20.0. Experimental factors that might potentially cause variability on the ion-pair complex formation were tested. Two factors, pH and volume of the BCG dye, were investigated from the preliminary works. A two-level full factorial design was applied with a number of runs equal to four (2^2^) experiments. The lower and higher values for each factor in the design are given in Table 1.

### 2.7. Method Validation

The developed method was validated according to the ICH (International Conference on Harmonization) guidelines [19] for its linearity, limit of detection (LOD), limit of quantification (LOQ), precision, and accuracy.

## 3. Results and Discussion

### 3.1. Method Development and Optimization

#### 3.1.1. Spectral Characteristics

**Figure 1:**
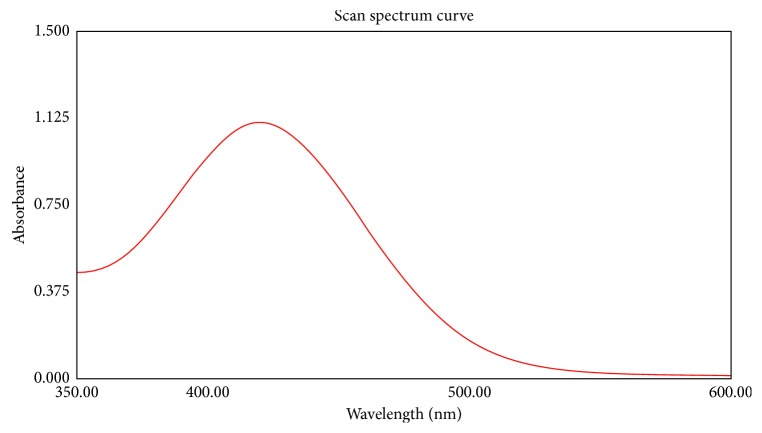
Absorption spectra of the miconazole-BCG ion-pair complex in chloroform.

The absorption spectra of the yellow-colored ion-pair complex formed from miconazole nitrate and BCG measured within the range of 350–600 nm against the blank are shown in Figure 1 with a maximum absorbance at 422 nm. The effect of various experimental parameters on the absorbance of the yellow-colored complex was studied.

#### 3.1.2. Effect of pH on the Ion-Pair Formation

The effect of pH on the complex formation was tested in the range of 4–6, and it was found that maximum complexation is achieved at pH 4. In addition, 2 mL of phthalate buffer provided maximum absorbances and reproducible results.

#### 3.1.3. Effect of Time

The effect of time on the stability of the yellow-colored ion-pair complex was examined until 3 h, and the complex was found to be stable up to 2 h at room temperature.

#### 3.1.4. Composition of the Complex

**Figure 2:**
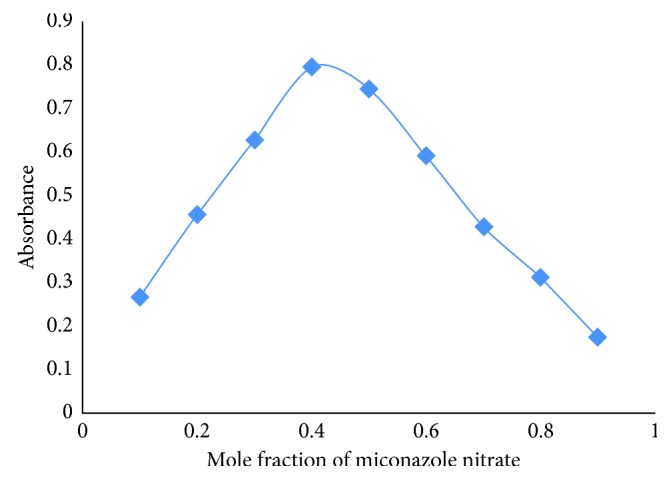
Job's method of the continuous variation plot for an ion-pair complex of miconazole-BCG in chloroform at 422 nm.

The stoichiometry of the drug-dye complex was investigated using Job's method of continuous variations (Figure 2). Maximum absorbance for the ion-pair complex was found at a mole ratio of 1 : 1.3 which showed the formation of 1 : 1 (miconazole : BCG) complex.

#### 3.1.5. Optimization of the Method

Optimization of the method was performed by experimental design as described in Section 2.6. Analysis of variance results for main factors and their interaction are shown in Table 2. The results revealed that both pH and volume of the BCG dye as well as their interaction had significant effect on the absorbance of the ion-pair complex (*p* < 0.05). The effect sizes of main factors and their interaction were estimated by eta squared, which is used to measure the effect size in analysis of variance models and has an interpretation similar to a coefficient of determination. From Table 2, eta squared of greater than 0.8 for pH and volume of the BCG as well as their interaction indicated their large effect sizes on the absorbance of the complex.

**Figure 3:**
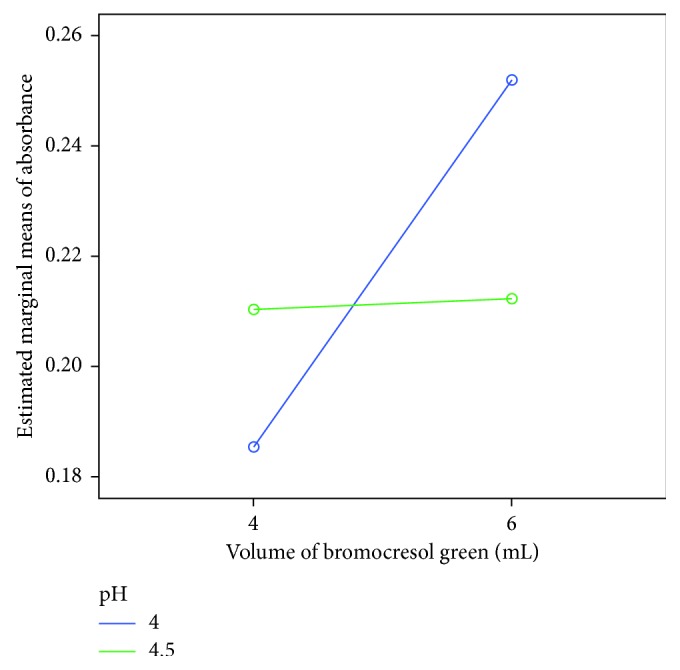
Interaction plot showing the influence of pH and volume of the BCG on absorbance.

The interaction plot revealed an interaction between pH and volume of the BCG, suggesting that the absorbance of the ion-pair complex significantly increased as the volume of the BCG changed from 4 mL to 6 mL at pH 4 (Figure 3). The significance of this interaction effect was confirmed by the results of the ANOVA (Table 2). Hence, pH 4 and 6 mL of BCG were found to be optimal for complete complexation and extraction based on the design of the experiment.

### 3.2. Method Validation

#### 3.2.1. Analytical Data

The proposed method was validated according to the ICH guidelines [19]. Under the optimized experimental condition, linearity of the absorbance was examined by analyzing a series of different concentrations of miconazole nitrate. The linearity of the calibration graph over the concentration range of 1–30 g/mL was proved by high coefficient of determination (*r*^2^) and the low value of the *y*-intercept of the regression equation. The statistical parameters calculated from the calibration graphs are given in Table 3. The molar absorptivity of the ion-pair complex was 2.285 × 10^4^ L/mol/cm which reveals the high sensitivity of the method. The limit of detection and the limit of quantification were calculated from a calibration curve constructed using solutions containing a miconazole nitrate in the range of detection limit, and the results are shown in Table 3.

#### 3.2.2. Precision

The precision of the proposed method was evaluated as intra- and interday precisions by calculating the percent relative standard deviation (% RSD). The intraday precision was estimated by analyzing six times the solution of a drug prepared according to the procedure, while the results of three consecutive days were used for the evaluation of intermediate precision. The determined % RSD for intraday ranged from 0.2 to 0.9 and to 1.2 for interday precision. The findings indicate the good precision of the method as the values of both precisions were <2%.

#### 3.2.3. Accuracy

The reliability and validity of the developed method were assessed by the standard addition technique. Known amounts of standard drug powders: 80%, 100%, and 120% of the test concentration, were added. Analysis was done in triplicate, and the accuracy of the method was evaluated as the percent recovery of the added amounts of the standard to the previously analyzed sample (Table 4). Percent recoveries ranged from 98.37% to 101.58%, which demonstrates that the matrices and/or excipients in cream and vaginal soft gelatin capsule products do not interfere in the quantification of miconazole nitrate.

#### 3.2.4. Application of the Method

The proposed method has been applied for the assay of miconazole nitrate in cream and soft gelatin capsule products. The samples were prepared in duplicates, and the absorbance was measured in triplicate. Average contents of 102.1% and 98.8% of the label claims were obtained for the cream and vaginal soft gelatin capsule samples, respectively. The findings were found to be in good agreement with the label claims for both formulations.

The proposed method is much easier than HPLC methods stipulated in Pharmacopeias [1, 15] and other existing analytical methods [3–11]. The reagents employed in the developed technique are cheaper and readily available, and the procedures do not involve any critical reaction conditions or tedious sample preparation.

## 4. Conclusion

The developed method is simple, precise, accurate, sensitive, and not time-consuming compared to chromatographic techniques. The good precision and accuracy of the method were supported by statistical parameters and recovery data. Therefore, it can be used for the routine analysis of miconazole nitrate in bulk and pharmaceutical preparations.

## Figures and Tables

**Table 1:** Parameter settings applied in the method optimization.

Parameter	Low value (−)	High value (+)
pH	4	4.5
Volume of the BCG (200 *μ*g/mL), mL	4	6

**Table 2:** Analysis of variance for pH and volume of the BCG and their interaction.

Source	Sum of squares	df	Mean square	*F* value	*p* value	Partial eta squared
Corrected model	0.007	3	0.002	506.222	0.001	0.995
Intercept	0.555	1	0.555	123,266.667	0.001	1.000
pH	0.000	1	0.000	35.852	0.001	0.818
Vol. BCG	0.004	1	0.004	785.852	0.001	0.990
pH ∗ vol. BCG	0.003	1	0.003	696.963	0.001	0.989
Error	3.600*E* − 005	8	4.500*E* − 006			
Corrected total	0.007	11				

df: degree of freedom.

**Table 3:** Optical characteristics of the proposed method.

Parameter	Value
*λ*_max_ (nm)	422
Beer's law limit (*μ*g/mL)	1–30
Molar absorptivity (L/mol/cm)	2.285 × 10^4^
LOD (*μ*g/mL)	0.0691
LOQ (*μ*g/mL)	0.2095
Linear regression equation (*Y* = *a* + *bc*)	
Slope (*b*)	0.0259
Intercept (*a*)	−0.0133
Coefficient of determination (*r*^2^)	0.9992

**Table 4:** Recovery study of miconazole nitrate from pharmaceutical formulations.

Formulation	Amount taken (*μ*g/mL)	Amount added (*μ*g/mL)	Amount found (*μ*g/mL)	% recovery
Cream	10	8	7.94 ± 0.77	99.3
	10	10	10.03 ± 0.69	100.3
	10	12	12.19 ± 0.45	101.6
Soft capsule	10	8	7.87 ± 0.84	98.4
	10	10	9.89 ± 0.72	98.9
	10	12	11.81 ± 0.93	98.4
